# Sodium Tetraphenylborate Displays Selective Bactericidal Activity against Neisseria meningitidis and N. gonorrhoeae and Is Effective at Reducing Bacterial Infection Load

**DOI:** 10.1128/AAC.00254-20

**Published:** 2021-01-20

**Authors:** Eve Bernet, Marthe Lebughe, Antony T. Vincent, Mohammad Mehdi Haghdoost, Golara Golbaghi, Steven Laplante, Annie Castonguay, Frederic J. Veyrier

**Affiliations:** a Bacterial Symbionts Evolution, Institut national de la recherche scientifique, Centre Armand-Frappier Santé Biotechnologie, Laval, Quebec, Canada; b Organometallic Chemistry Laboratory for the Design of Catalysts and Therapeutics, Institut national de la recherche scientifique, Centre Armand-Frappier Santé Biotechnologie, Laval, Quebec, Canada; c Institut national de la recherche scientifique, Centre Armand-Frappier Santé Biotechnologie, Laval, Quebec, Canada

**Keywords:** *Neisseria gonorrhoeae*, *Neisseria meningitidis*, tetraphenylborate, antibiotics, antibiotic resistance

## Abstract

Neisseria meningitidis and Neisseria gonorrhoeae, two highly related species that might have emerged from a common commensal ancestor, constitute major human threats. Vaccines are available to prevent N. meningitidis infection, whereas there are only a limited number of antibiotics available for N. gonorrhoeae. Unfortunately, some strains of these species are rapidly evolving and capable of escaping human interventions. Thus, it is now urgent to develop new avenues to fight these bacteria. This study reports that a boron-based salt, sodium tetraphenylborate (NaBPh_4_), displays high bactericidal activity and remarkable specificity against N. meningitidis and N. gonorrhoeae. Other closely related commensal species such as Neisseria lactamica, which is found in the normal flora of healthy individuals, were found to be less affected even at 5-fold higher doses of NaBPh_4_. This specificity was further observed when much lower sensitivity was found for more distant *Neisseriaceae* species (such as Neisseria elongata or Kingella oralis) and completely unrelated species. Significant boron uptake by N. meningitidis cells was observed after incubation with 5 μM NaBPh_4_, as measured by inductively coupled plasma mass spectrometry, suggesting that this drug candidate’s target(s) could be located intracellularly or within the cell envelope. Furthermore, mutants with slightly decreased susceptibility displayed alterations in genes coding for cell envelope elements, which reduced their virulence in an animal model of infection. Finally, a single dose of NaBPh_4_ resulted in a significant reduction in bacterial burden in a mouse model of N. meningitidis bacteremia. Although numerous boron-containing species were previously reported for their complex biological activities, the observation of this narrow selectivity is unprecedented and of potential importance from a therapeutic standpoint.

## Abstract (precis)

Neisseria meningitidis and Neisseria gonorrhoeae, two highly related species that might have emerged from a common commensal ancestor, constitute major human threats. Vaccines are available to prevent N. meningitidis infection, whereas there are only a limited number of antibiotics available for N. gonorrhoeae. Unfortunately, some strains of these species are rapidly evolving and capable of escaping human interventions.

## INTRODUCTION

The *Neisseria* genus comprises bacteria that have been mainly isolated from either mammalian hosts (such as humans, dolphins, and sea lions) or nonmammalian hosts (such as iguanas), and only two species are pathogenic, i.e., Neisseria meningitidis and Neisseria gonorrhoeae. These species are closely related and could be considered subspecies (1). N. meningitidis asymptomatically resides in the human nasopharynx and, under some circumstances that are not yet understood, can enter the bloodstream and cause severe septicemia (leading to purpura fulminans) and/or cerebrospinal meningitis (2). N. gonorrhoeae is the etiological agent of gonorrhea and is transmitted across urogenital tissues. Infections occur in male urethra, in female lower genital tract mucosae (primarily the cervix), and less frequently in extragenital mucosal sites, including the oropharynx, the anorectum, and the eyes of neonates, with severe side effects that often result in blindness (3). Less commonly, the bacteria may enter the bloodstream, resulting in a disseminated infection with septicemia or meningitis (3).

Currently, preventive or therapeutic strategies are used against these two bacteria. For instance, vaccination against N. meningitidis is efficient in controlling the spread of some invasive strains (such as the serogroups A, C, W, and Y) with the use of oligosaccharide-based conjugate vaccines (4). More recently, a protein-based vaccine has been employed to prevent the spread of serogroup B strains. To treat the disease, an intravenous (i.v.) or intramuscular injection of penicillin or ceftriaxone is commonly used (5). Other antibiotics employed for the treatment of meningococcal diseases include chloramphenicol, fluoroquinolones, and meropenem (5). Despite this, there is evidence that reduced susceptibility to antibiotics is increasing worldwide (6). This disease still affects approximately 1 million people and kills around 0.2 million every year, in addition to damage from sequelae. Consequently, in 2018 the World Health Organization (WHO) introduced the worldwide initiative Defeating Meningitis by 2030, which aims at finding key solutions to decrease the devastating outcomes of this disease. In comparison, the situation for N. gonorrhoeae is a more major worldwide concern (7). There currently exists no vaccine for this disease, and treatments are mainly based on broad-spectrum antibiotics such as ceftriaxone and azithromycin (3). Unfortunately, N. gonorrhoeae has an extremely high mutation rate and easily exchanges DNA with other species (8). Therefore, strategies for combating this disease need to be constantly updated as a result of this bacterium’s exceptional capacity to change and to adapt. For example, the Public Health Agency of Canada has recently reported the occurrence of N. gonorrhoeae resistance to several antibiotics, including macrolides (such as azithromycin) and cephalosporins (9). As a consequence, there is a high likelihood of emerging extensively drug-resistant (XDR) gonococci that would be untreatable (10, 11). The U.S. Centers for Disease Control and Prevention (CDC) recently urged the scientific community to continuously monitor antibiotic resistance in N. gonorrhoeae, and the WHO has included this species in its global priority list of antibiotic-resistant bacteria to guide discovery and development of new antibiotics (12).

In several cases, nonpathogenic commensal symbionts participate in the protection of the host against pathogenic species. This is also the case for pathogenic *Neisseria* species. For example, a study showed that carriage of Neisseria lactamica has a protecting effect against N. meningitidis infections (13). Taking this factor into account, we have undertaken a screen for antibacterial molecules with an ability to specifically target N. meningitidis and N. gonorrhoeae pathogens but with a minimal effect on the commensal flora. Using a luminescent N. meningitidis strain, we first undertook an anti-*Neisseria* drug screen with a library of molecules. Although several compounds were found to display significant activity, a specific group of molecules with a common anion (BPh_4_^−^) were found to be highly effective at killing pathogenic *Neisseria* species without considerably affecting other bacteria, including other species of the same genus (14). Although numerous boron-containing compounds have already been reported for their diverse biological activities, the specificity for pathogenic *Neisseria* species observed in this study has rarely been achieved for antibiotics and therefore represents an attractive and promising therapeutic avenue. Here, we report the *in vitro* activity of NaBPh_4_ against the two aforementioned pathogens and demonstrate the *in vivo* ability of this lipophilic anion to reduce the bacterial burden during experimental bacteremia using a mouse model of infection.

## RESULTS

### Library screening for the identification of compounds with the ability to inhibit the growth of N. meningitidis.

**FIG 1:**
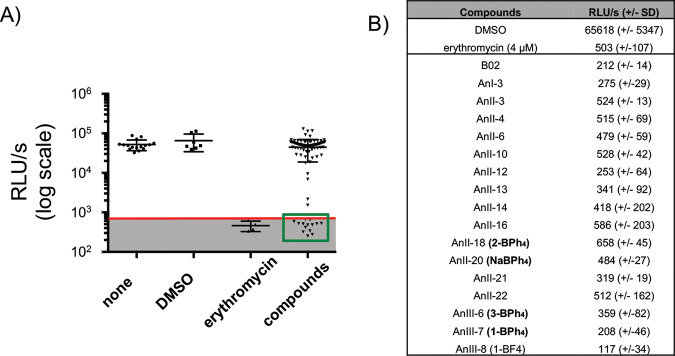
Library screening for the identification of compounds with the ability to inhibit the growth of N. meningitidis. (A) Effects of a subset of the compounds, tested at 100 μM, on the 16-h growth of N. meningitidis measured using a luciferase-based assay. The background noise (gray shaded area) was set at 800 RLU/s based on replicate measurements of growth in presence of erythromycin. (B) Table showing mean RLU per second obtained after 16 h of growth for the controls (1% DMSO or 4 μM erythromycin) and active compounds tested at 100 μM. The means represent a minimum of three independent replicates.

**FIG 2:**
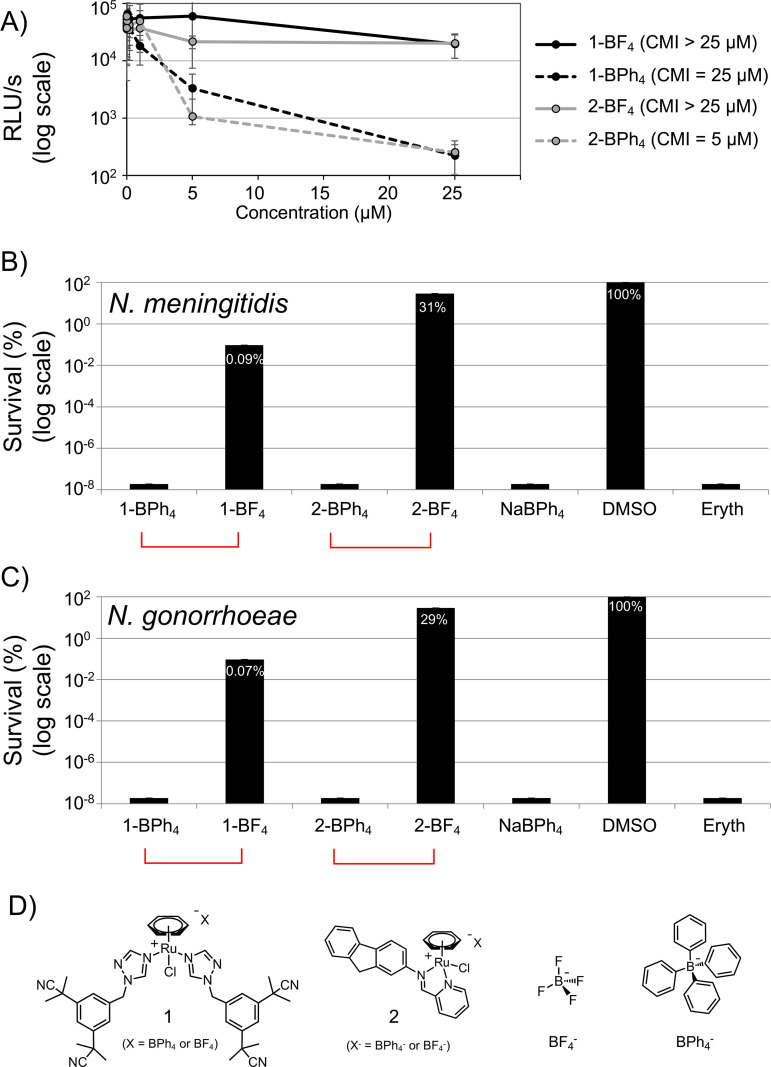
BPh_4_^−^ (and BPh_4_^−^-based compounds) efficiently kills N. meningitidis and N. gonorrhoeae. (A) Effects of increased concentrations of BPh_4_^−^ (and BPh_4_^−^-based compounds) on the 16 h growth of N. meningitidis measured using a luciferase-based assay. (B and C) Survival of N. meningitidis (B) and N. gonorrhoeae (C), expressed as a percentage of the 1% DMSO treatment condition, after 3-h treatment with 50 μM solutions of the compounds. Each bar represents the average of three independent measurements, and error bars represent the standard deviations. (D) Structures of compounds 1 and 2 and their BF_4_^−^ and BPh_4_^−^ counterions.

To screen molecules for their antibacterial activity, a luminescent system was used, as previously employed for other bacterial studies (15). A clinical isolate of N. meningitidis (isolate LNP24198) expressing luciferase (LuxABCDE) under the control of the constitutive promoter *porB*p was used, as described elsewhere (16). Of note, the correlation between CFU counts and luminescence was previously validated (16, 17). Approximately 2,500 compounds, mostly from a well-curated library of fragment compounds (18), were screened to identify candidates with the ability to completely inhibit the growth of N. meningitidis at 100 μM after 16 h at 37°C. As presented in Fig. 1A, showing a sample of these results, only 17 compounds (approximately 0.7% of the library) met this criterion. For these experiments, erythromycin was used as a positive control (Fig. 1B) (<800 RLU/s, the background luminescence, was found to be the largest value measured in 10 replicates with erythromycin at 3.5 μM, which is known to inhibit all growth of N. meningitidis). Stock solutions for all tested compounds were prepared in dimethyl sulfoxide (DMSO), and the final DMSO concentration was kept at 1%, which does not affect the growth of N. meningitidis (Fig. 1A). Interestingly, 4 of the 17 active compounds harbored a tetraphenylborate anion (BPh_4_^−^) and were found to be the only molecules from the screened library to include this moiety (e.g., 1-BPh_4_ and 2-BPh_4_ [19, 20]) (Fig. 2D). An investigation of the effect of the BPh_4_^−^ moiety was then pursued.

### BPh_4_^−^ efficiently kills N. meningitidis and N. gonorrhoeae.

**FIG 3:**
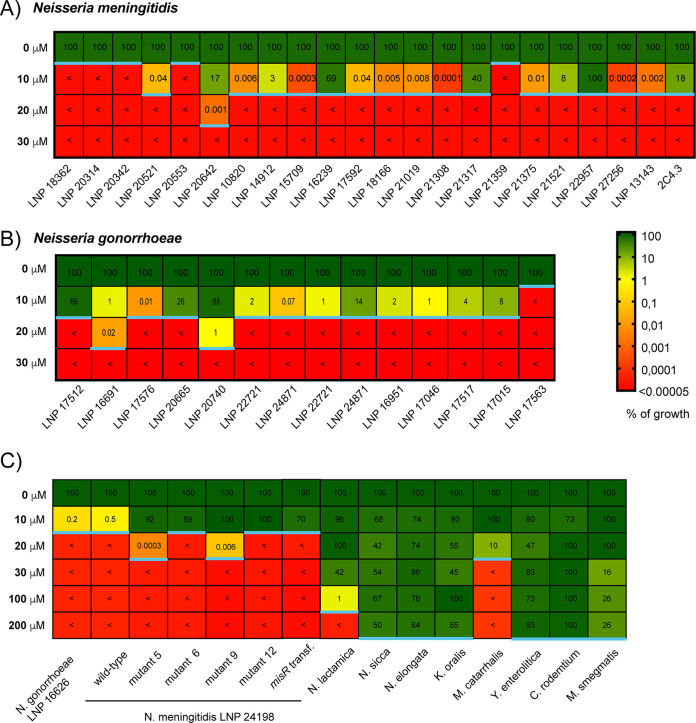
MICs measured by agar dilution assay. The tables show the percent growth of representative N. meningitidis strains (A) and N. gonorrhoeae strains (B) (21) and a panel of other species (C). The results are color coded as indicated, and < represents a score below our limit of detection (0.00005%). The blue line represents the MIC for the corresponding species (growth of <0.00005%). Each number represents the average of three independent measurements.

The library screen allowed the identification of candidates with considerable bacteriostatic activity but did not provide information about their bactericidal effects. Bactericidal activity was assessed for the three most active compounds identified from our initial screen, which all included a BPh_4_^−^ moiety in their structures. N. meningitidis was treated with 50 μM each compound, and the percentage of surviving cells (compared with the control [1% DMSO]) was measured using serial dilutions and CFU counts. After 3 h, no live bacteria could be detected, as seen in Fig. 2B. To determine whether the bactericidal effect could be observed with other *Neisseria* pathogens, a clinical isolate of. N. gonorrhoeae (isolate LNP16626 [21]) was also treated with compounds at 50 μM using the same assay. Again, no viable cells were detected after 3 h, suggesting that BPh_4_^−^ is also toxic for N. gonorrhoeae (Fig. 2C). Of note, we tested several strains of N. meningitidis and N. gonorrhoeae and observed similar results using a standard agar dilution assay (Fig. 3A and B). Given these notable observations, we concluded that BPh_4_^−^ clearly displays bactericidal activity against N. meningitidis and N. gonorrhoeae.

### BF_4_^−^, a tetrahedral boron anion analogue, does not kill N. meningitidis and N. gonorrhoeae.

To verify that BPh_4_^−^ is the moiety with an ability to eradicate the two pathogens of the *Neisseria* genus, both bacteria were exposed to analogues of 1-BPh_4_ and 2-BPh_4_ at 50 μM. We used 1-BF_4_ and 2-BF_4_, for which the only structural difference lies in the nature of their counterion (BF_4_^−^ versus BPh_4_^−^). Under those conditions, the two analogues with BF_4_^−^ counterion were found to be much less active than their BPh_4_^−^ counterparts in inhibiting the growth of N. meningitidis (Fig. 2A) or in killing both pathogens (Fig. 2B and C).

### Several BPh_4_^−^ salts (Na^+^, K^+^, and NH_4_^+^) are toxic to N. meningitidis.

**FIG 4:**
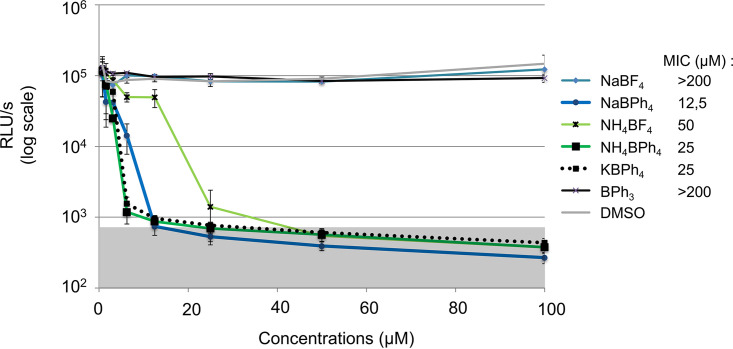
Toxicity of several BPh_4_^−^ salts (Na^+^, K^+^, and NH_4_^+^) to N. meningitidis. Concentration-dependent growth inhibition and MICs for different BPh_4_^−^ and BF_4_^−^ salts after 16 h of growth are shown. Each point represents the average of three independent measurements, and error bars represent the standard deviations. The background noise (gray shaded area) was set at 800 RLU/s based on replicate measurements of growth in presence of erythromycin.

To rule out any effects on the antimicrobial activity due to the nature of the cation, we measured the growth of N. meningitidis after a 16-h exposure to different concentrations of various BPh_4_^−^ and BF_4_^−^ salts with different cations. These results are presented in Fig. 4. From this assay, we calculated the MIC (the lowest concentration that prevents visible bacterial growth [here, <800 RLU/s]). All BPh_4_^−^ salts tested (sodium, ammonium, and potassium) were highly and similarly active. As observed for analogous BPh_4_^−^/BF_4_^−^ complexes previously tested, BPh_4_^−^ salts were found to be more active than their BF_4_^−^ counterparts (sodium and ammonium).

### Only N. meningitidis and N. gonorrhoeae are completely killed after a 3-h exposure to NaBPh_4_.

**FIG 5:**
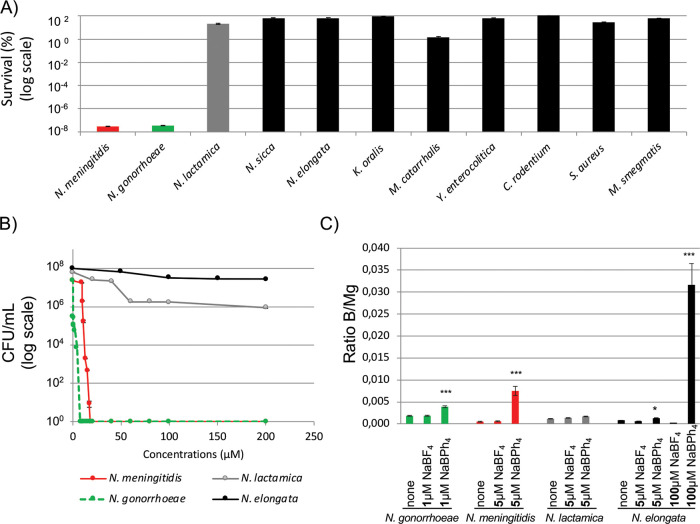
Selectivity of NaBPh_4_ toxicity and B uptake. (A) Percent survival of a panel of strains exposed to a 50 μM solution of NaBPh_4_ for 3 h. (B) Concentration-dependent survival (expressed in total CFU) for four selected strains. (C) ICP-MS quantification of the B uptake (normalized to Mg content) for N. gonorrhoeae, N. meningitidis, N. lactamica, and N. elongata grown in rich medium with or without NaBPh_4_ or NaBF_4_. The data are expressed as B/Mg ratios. Each bar represents the average of three independent measurements, and error bars represent the standard deviations. ***, *P* < 0.001; *, *P* < 0.05.

To assess the selectivity of the observed BPh_4_^−^ toxicity, we measured the MICs (the lowest concentration that prevents visible bacterial growth [here, our limit of detection, 0.00005%]) of several strains using a standard agar dilution assay (Fig. 3C). Unexpectedly, the closely related *Neisseria* species N. lactamica (MIC of 200 μM) was clearly not found to be as susceptible as the pathogenic species N. meningitidis and N. gonorrhoeae (MICs of <10 μM). This result was surprising, considering their close phylogenetic proximity (22). Other *Neisseria* strains, such as Neisseria sicca (MIC of >200 μM) and Neisseria elongata (MIC of >200 μM) were also tested and were not found to be susceptible to NaBPh_4_. Kingella oralis (MIC of >200 μM), another member of the *Neisseriaceae* family, was also noted to be resistant to NaBPh_4_, suggesting that for some unknown reason(s) the pathogenic species N. meningitidis and N. gonorrhoeae have selective sensitivity to this compound. . Other Gram-negative species, such as Yersinia enterocolitica (MIC of >200 μM), Citrobacter rodentium (order *Enterobacterales*) (MIC of >200 μM), and Moraxella catarrhalis (order *Pseudomonadales*) (MIC of 30 μM), as well as Gram-positive species such as Staphylococcus aureus (MIC of >200 μM) and Mycobacterium smegmatis (phylum *Actinobacteria*) (MIC of >200 μM), were tested. Again, they were all found to be much more resistant to NaBPh_4_ than N. meningitidis and N. gonorrhoeae. Of note, we also measured the percent survival of these species after they were exposed to 50 μM NaBPh_4_ for 3 h in liquid medium (Fig. 5A), and we again observed that N. meningitidis and N. gonorrhoeae were more sensitive to NaBPh_4_ than all of the other species tested.

We next assessed in liquid medium the minimum bactericidal concentration (MBC), which is the concentration necessary to kill >99.9% of the bacterial population after a 3-h exposure to NaBPh_4_. CFU counts for the four species tested, i.e., N. elongata, N. lactamica, N. meningitidis, and N. gonorrhoeae, are presented in Fig. 5B. N. meningitidis (which is capsulated) and N. gonorrhoeae were clearly found to be the species most sensitive to NaBPh_4_ exposure, with MBCs of 13 μM and 4 μM, respectively, whereas N. lactamica and N. elongata were found to have lower sensitivity to this salt, with MBCs above 200 μM (the highest concentration tested).

### Cellular boron levels are higher in N. meningitidis and N. gonorrhoeae than in other *Neisseria* species after growth in agar medium containing NaBPh_4_.

In order to gain more insight into the interaction of BPh_4_^−^ with different *Neisseria* bacteria, cellular boron levels (normalized with magnesium) were assessed by inductively coupled plasma mass spectrometry (ICP-MS), as described previously (21), for four strains (N. elongata, N. lactamica, N. meningitidis, and N. gonorrhoeae) (Fig. 5C). Of note, this assay allowed the measurement of cellular boron incorporation but did not allow a distinction between its internalization within the cell envelope or the cytoplasm. Also, due to the greater sensitivity of N. gonorrhoeae to BPh_4_^−^, a lower concentration (1 μM versus 5 μM) of NaBPh_4_ (or NaBF_4_) was used for boron internalization experiments involving this strain. In comparison with an untreated control (growth on GCB agar), a >2-fold increase in boron cellular levels was noted when N. gonorrhoeae was exposed to 1 μM NaBPh_4_, whereas cellular boron levels were found to be similar to the control when the same strain was exposed to 1 μM NaBF_4_. When N. meningitidis was exposed to 5 μM NaBPh_4_, a 7-fold increase in cellular boron levels was observed, compared to an untreated control (growth on GCB agar), whereas boron cellular levels were found to be similar to the control levels when the same strain was exposed to 5 μM NaBF_4_. Interestingly, no significant boron internalization was found (compared to controls) for other *Neisseria* species tested with 5 μM NaBPh_4_ and NaBF_4_. This suggests that the internalization of boron is significant for treatment of the two pathogenic species with NaBPh_4_, whereas that is not the case for the other tested species.

To gain additional insights into the ability of BPh_4_^−^ and BF_4_^−^ to penetrate less susceptible species, N. elongata was exposed to 100 μM NaBPh_4_ and NaBF_4_ (Fig. 5C). Boron levels were found to be 61-fold higher for NaBPh_4_, indicating greater penetration of the BPh_4_^−^ ion. Interestingly, despite the high level of boron uptake, 100 μM NaBPh_4_ did not inhibit the growth of N. elongata (Fig. 5B).

### N. meningitidis naturally occurring mutations lead to only slight decreases in sensitivity.

**FIG 6:**
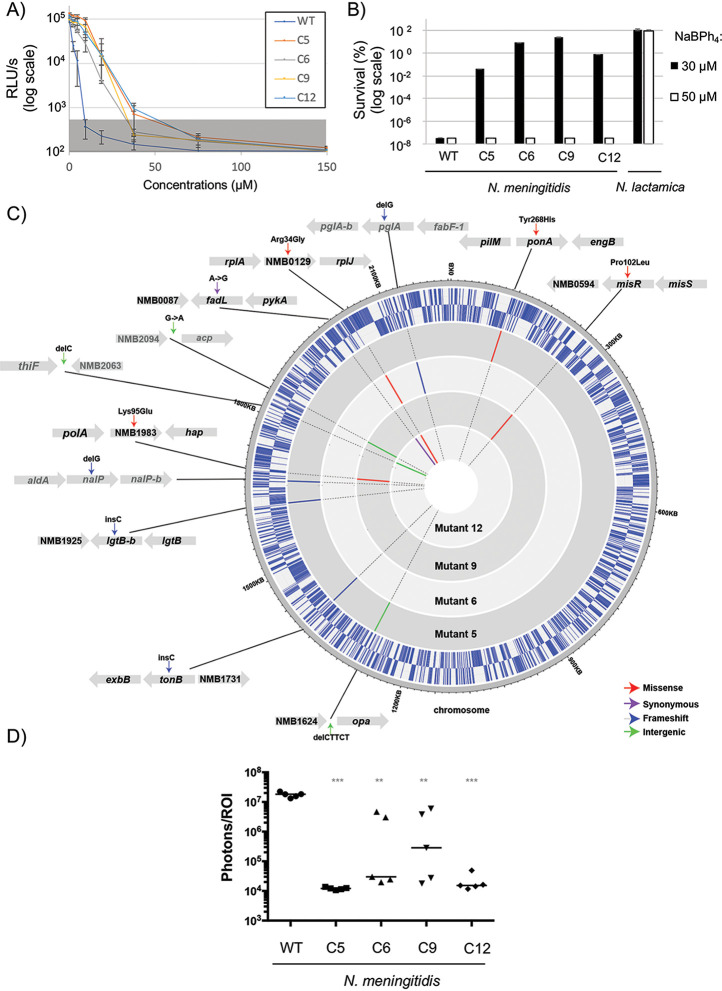
Mutations in independently isolated clones with decreased sensitivity. (A) Concentration-dependent growth inhibition of different clones in contact with NaBPh_4_. The background noise (gray shaded area) was set at 800 RLU/s based on replicate measurements of growth in presence of erythromycin. (B) Percent survival of wild-type N. lactamica and N. meningitidis and the mutants with decreased sensitivity, after 3-h treatment with a solution of 30 or 50 μM NaBPh_4_. In panels A and B, each point represents the average of three independent measurements, and error bars represent the standard deviations. (C) Graphical representation of N. meningitidis mutations detected in each clone with decreased NaBPh_4_ sensitivity. External circles represent the wild-type genome positions with genes (in blue) organized on the basis of their orientation (first circle for positive orientation and second circle for negative orientation). Dark gray represents mutations harbored by 100% of the cell population, whereas light gray represents a mixed population. (D) Bacterial burden in the mouse coinfection model, measured after 24 h, for wild-type (WT) N. meningitidis and the different mutants with decreased sensitivity. Each bar represents the median measurement for five independent mice. ***, *P* < 0.001; **, *P* < 0.01.

We attempted to isolate resistant clones of N. meningitidis by growing 2 × 10^8^ bacteria on GCB plates containing 17.5 μM NaBPh_4_. After multiple attempts, we were able to isolate only four clones that grew under these conditions (estimated rate of clones with decreased sensitivity of around 1 × 10^−9^). The effect of the NaBPh_4_ concentration on the 16-h growth was measured as done previously for wild-type N. meningitidis. As seen in Fig. 6A, the four clones had decreased sensitivity to NaBPh_4_, with MICs measured in liquid medium that ranged from 37.5 to 75 μM (2 to 3 times more than the wild-type MIC). We next measured the percentage of bacterial survival when the clones were exposed to 30 or 50 μM NaBPh_4_ for 3 h. As seen in Fig. 6B, all clones showed better survival rates than the wild-type strain with 30 μM. Therefore, their MBCs (values of around 50 μM) were higher to that for the wild-type strain (13 μM). Four mutants were sequenced, and multiple mutations were observed in each (see Table S1 in the supplemental material). Mutations were verified by Sanger sequencing (data not shown) because they could correspond to false-positive results (due to an on/off switch and the Neisseria meningitidis intrinsic mutation rate). In this sense, some mutations (indicated in Fig. 6C in light gray, i.e., *nalP*, *pglA*, and, in the intergenic regions, *thiF-nmb2063* and *nmb2094-acp*) showed conflicting chromatograms that demonstrated that the mutation was not harbored by 100% of the cell population, as is expected to be the case, independently of the susceptibility to NaBPh_4_, for genes that are subject to phase variation (23). On the other hand, the majority of the mutations that were confirmed for the entire population (dark gray in Fig. 6C) were located in genes coding for proteins implicated in cell envelope permeability. Of note, to the best of our knowledge, the genes (except *opa* and *lgtB*) with confirmed mutations, namely, *misR*, *ponA1*, *tonB*, *fadL*, *nmb0129* (obtained twice), and *nmb1983*, have not been shown to be subject to phase variations. Finally, as a control, we attempted to use genomic DNA transformations to confirm the effects of such mutations. We were able to recover clones with decreased sensitivity with the *misR* Pro102Leu mutation (Fig. 3C). This suggests that multiple mechanisms may be implicated but an alteration of the cell envelope permeability of N. meningitidis (via a MisR regulon misregulation) could affect NaBPh_4_ sensitivity, as observed for other antibiotics (24).

### Fitness cost for the slightly increased resistance.

In light of the mutations obtained in our evolved less sensitive clones, we hypothesized that these clones would experience a defect in virulence. Using a coinfection model (influenza virus and N. meningitidis) of mice bacteremia, we measured the bacterial burden during infection and compared it with that of the wild-type parental strain (Fig. 6D). For this, we used a H1N1 virus coinfection model that was described previously (25, 26) and that was shown to increase the pathophysiology and to generate a N. meningitidis brain infection (25, 26). We observed defects in virulence for all of the clones, which were maximally visible at 24 h. The most attenuated clone was found to be c5 (with mutations in *tonB* and *ponA*, among others), which was completely cleared after 24 h.

### NaBPh_4_ can be used to attenuate N. meningitidis infections in mice.

**FIG 7:**
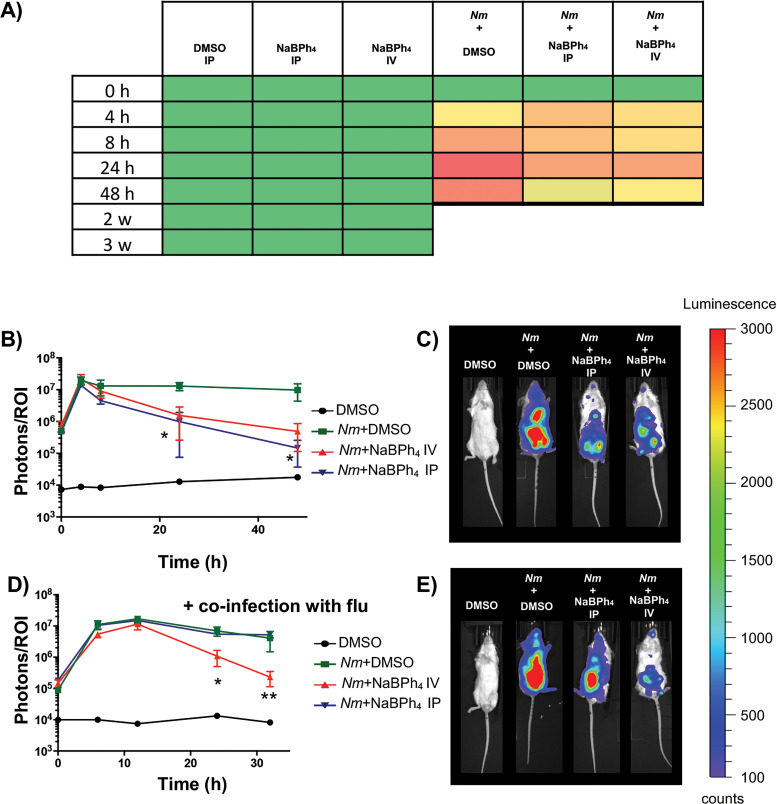
NaBPh_4_ can be used to treat a pathogenic *Neisseria* disease. (A) Average health state of each group, measured after injection of 100 μl of a solution of vector (1% DMSO i.p.) or NaBPh_4_ (i.p. or i.v.), with or without N. meningitidis infection. Clinical signs were scored as followed: green, no sign; yellow, +; orange, ++; dark orange, +++; red, ++++. (B) Time course of N. meningitidis burden (measured using luminescence) in a mouse model of bacteremia with or without treatment. Each point represents the median measurement of total photon counts in a defined ROI for three to five independent mice. (C) Representative images of mice 24 h postinfection with or without treatment. (D) Time course of N. meningitidis burden in a mouse model of coinfection with influenza virus with or without treatment. Each point represents the median measurement for five independent mice. Experiments without influenza virus were done in duplicate, and results were confirmed with a coinfection experiment. (E) Representative images of mice 24 h postcoinfection with or without treatment. **, *P* < 0.01; *, *P* < 0.05.

In order to test whether NaBPh_4_ could be used to cure *Neisseria* pathogen infections, we used several previously described mouse infection models of N. meningitidis bacteremia (16, 25, 26). We tested a single injection (either an intraperitoneal [i.p.] injection or an i.v. injection), 2 h postinfection, of 100 μl of 20 μM NaBPh_4_ solution (0.7 μg of NaBPh_4_/mouse). This concentration was chosen because it is close to the *in vitro* MIC. As a prerequisite, we first performed a toxicity test on five mice per group and measured the general state of health by measuring five criteria (activity/lethargy, posture [such as hunched or prostrated], eye abnormality, ruffled fur, and social behaviors). The noninfected mice treated with NaBPh_4_ did not show any visible sign of general toxicity (for the compound or the vehicle) for 4 weeks (the duration of this toxicity assessment), as seen in Fig. 7A. When infections were performed, treatment with NaBPh_4_ led to significant reductions, maximally visible after 24 h, of the bacterial burden, compared to infected mice injected with the vehicle only (0.1% DMSO in phosphate-buffered saline [PBS]), as seen in Fig. 7B and C. In addition, after 24 h, treated infected mice (either i.p. or i.v. injection) showed decreased symptoms of the disease (such as squinted eyes, ruffled fur, and lethargy) (Fig. 7A). Of note, for ethical reasons, we used a nonlethal dose of N. meningitidis infection. We subsequently performed a coinfection with influenza virus, as described above. In that case, we observed that NaBPh_4_ led to a significant reduction of the bacterial burden only in the case of i.v. injection. Of note, all mice were sacrificed after 32 h of infection, as our critical point of health was reached.

## DISCUSSION

In the present study, we report a lipophilic, boron-based, tetrahedral anion, BPh_4_^−^, which is highly toxic for the two pathogenic *Neisseria* species, namely, N. meningitidis and N. gonorrhoeae. Tetrahedral borate anions were previously reported to display interesting antimicrobial activities, as is the case, for instance, for tartrolon, borophycin, boromycin, and aplasmomycin (27). Although BPh_4_^−^ anions are commonly used in chemistry, notably as counterions for cationic metal complexes, the study of their biological activity has been largely overlooked. Notably, it was found that BPh_4_^−^ can be strongly absorbed at the surface of lipid bilayer membranes (30) and can increase the membrane permeability of some penetrating cations in Staphylococcus aureus (31) and in mitochondria (32). In addition, sodium tetraphenylborate was found to be an inhibitor of NO_2_^−^ oxidation in bacteria, and it was suggested that this compound may also disrupt the proton motive force, as shown using electron transport particles prepared from Nitrobacter winogradskyi (33). Therefore, we could hypothesize a mechanism of action in which BPh_4_^−^ salts destabilize the membrane and/or some membrane-associated processes of pathogenic *Neisseria* strains, which could lead to their lysis.

Interestingly, we have shown that this sensitivity is a unique property of pathogenic *Neisseria* species in both liquid culture (Fig. 5) and solid medium (Fig. 3). We found that the toxic dose of NaBPh_4_ was around 13 μM for N. meningitidis and 4 μM for N. gonorrhoeae. For other *Neisseria* species, particularly N. lactamica, which is closely phylogenetically related to the two pathogenic *Neisseria* species, this effect was not observed even at a 10-fold higher NaBPh_4_ concentration. ICP-MS experiments have shown that boron is more significantly imported in the cells of pathogenic species. Nevertheless, nonpathogenic species harbor similar, if not superior, cellular levels of boron when grown on 200 μM NaBPh_4_, without showing any signs of toxicity. Therefore, the selectivity of the NaBPh_4_ may arise from a combination of its more efficient uptake and its higher cellular toxicity for pathogenic species. Of note, the exact nature of the toxic compound remains to be determined, because NaBPh_4_ can be modified and this putative alteration might differ from one *Neisseria* strain to another. The membranes of *Neisseriaceae* species have numerous unique properties, and pathogenic *Neisseria* species have also evolved their particularities. One simple explanation for the specificity could be that nonpathogenic *Neisseria* species harbor an unknown NaBPh_4_ exporter or that pathogenic bacteria harbor a NaBPh_4_ importer. To date, it has not been shown that some borates can be exported by bacteria; however, studies have shown that some can potentially play a role in the iron transport system of bacteria. For instance, B(OH)_4_^−1^ can act as a synergistic anion for the periplasmic FbpA during Fe^3+^ transport in Marinobacter algicola cells at oceanic pH 8 (34). Of note, FbpA is present in pathogenic *Neisseria* species and in N. lactamica but not in the other *Neisseria* strains tested, making the mechanism of selectivity uncertain. On the other hand, *Neisseria* species harbor lipooligosaccharides (LOSs), which are structurally related to the lipopolysaccharides of enteric Gram-negative bacteria but lack the longer repeating O antigens (35). These LOSs are components of the outer leaflet of the outer membrane. The variable substituents and modifications (such as phosphorylation and phosphoethanolamine) of the LOSs make them highly heterogeneous even in the same clonal population of a neisserial species (36). To explain the difference in sensitivity between pathogenic *Neisseria* species and other *Neisseria* species, one could consider lipid A pyrophosphorylation and phosphoethanolaminylation, which have been described in pathogenic species, whereas commensal species lack *lptA* and are more sensitive to positively charged polymyxin B (37). Again, this difference may not be responsible for the selectivity of BPh_4_^−^, because BPh_4_^−^-resistant N. lactamica species contain a functional LptA (37). Therefore, it remains unknown why pathogenic *Neisseria* species are more sensitive and whether some uniquely evolved cell envelope properties allow the specific penetration and killing by NaBPh_4_. As a first clue, we generated a limited number of mutants that showed decreased sensitivity to the compound. As expected, we observed that each of the mutants harbored several mutations, with the majority of them being linked to the cell envelope. We could cite, for example, MisR (Pro102Leu), which has been shown to be implicated in membrane permeability (24), PonA (Tyr268His), which is a penicillin-binding protein (PBP) (PBP1) implicated in peptidoglycan synthesis (38), and TonB (frameshift mutation), which provides energy to iron transporters. In two of the four clones, we also confirmed a mutation in the gene NMB0129, which is located in a locus with *rplJ*, *rplL*, and *rpoB*. Unfortunately, this gene encodes a small protein conserved in *Neisseria* species but with an unknown function. When we transformed genomic DNA, we obtained clones only for the MisR mutation (data not shown). Overall, this suggests that, except for the MisR mutation, which is sufficient by itself, multiple mutations are necessary for this slight resistance. Unfortunately, due to the highly unstable nature of the N. meningitidis genome, we cannot rule out the possibility that some of these mutations are independent of the phenotype observed.

Although the mechanisms of action and the reasons for the selective antimicrobial activity of NaBPh_4_ are not completely understood, we demonstrated its *in vivo* potential to treat a *Neisseria* infection. We used mouse models of N. meningitidis bacteremia and showed that, using i.v. injection (0.7 μg of NaBPh_4_/mouse), the bacterial load could be decreased even in the case of coinfection with influenza virus. It is interesting to note that all of the less sensitive mutants harbored drastically decreased virulence. This clearly suggests that there is a cost for the bacteria to acquire intrinsic resistance to this compound.

To conclude, this study demonstrates that the BPh_4_^−^ framework could be exploited for the development of novel families of antibacterial compounds for the two devastating diseases discussed above, including its introduction into the structure of gold standard antibiotics such as ceftriaxone. In particular, such compounds could play an important role in the design of original approaches to thwart the emergence of XDR gonococci (10, 11).

## MATERIALS AND METHODS

All of the protocols reported for biological studies were approved by the Institutional Research Ethics Committee of the INRS.

### Bacterial strains and culture conditions.

All *Neisseriaceae* strains and Moraxella catarrhalis were grown in GCB agar medium with Kellogg supplements. Other strains were grown at 37°C in Luria-Bertani medium (Difco). When required, the antibiotic erythromycin (3 μg/ml) was added. S. aureus (strain 33592), N. elongata subsp. *glycolytica* (strain 29315), N. lactamica (strain 23970), *N. sicca* (strain 29256), M. smegmatis (strain 700084), and *K. oralis* (strain 51147) were obtained from the American Type Culture Collection (ATCC). Y. enterocolitica DSM23249 was purchased from the Deutsche Sammlung von Mikroorganismen und Zellkulturen (DSMZ) GmbH. M. catarrhalis LNP18103, N. meningitidis LPN24198, and N. gonorrhoeae LNP16626 and other LNP isolates were obtained as a donation from Muhamed-Kheir Taha from the Centre National de Reference des Meningocoques (Institut Pasteur, Paris, France), whereas C. rodentium DBS100 was obtained as a donation from Hervé le Moual (McGill University). An A/Puerto Rico/8/1934(H1N1) influenza virus preparation at 2 × 10^5^ PFU/ml, made from mouse lung homogenates in 30% glycerol and stored at −80°C, was obtained from Maziar Divangahi (McGill University).

### N. meningitidis luminescent strain growth assay (16 h).

To perform the library screening (Fig. 1) or to compare the activity of the different boron-containing salts (Fig. 2A, 4, and 6A), the amount of light produced after 16 h of growth for a N. meningitidis luminescent strain was measured. Contrary to optical density (OD) readings, which can be misleading due to the absorbance of dead cells, this measurement is directly correlated with the amount of live cells, because the half-lives of the luciferase and its substrate are limited and the emission of light thus does not continue after the death of the cells. To perform this assay, N. meningitidis (isolate LNP24198) expressing luciferase (LuxABCDE) under the control of the constitutive promoter *porB*p was grown overnight in GCB agar medium, and a cell suspension corresponding to an OD_600_ of 0.01 was subsequently prepared. In parallel, fresh stock solutions of the compounds in DMSO were prepared (100×, with final concentrations being indicated for the different figures), and 1.8 μl of the corresponding solution per well was added to 96-well plates. In each well, 178.2 μl of bacterial suspension was subsequently added. Bacteria were allowed to grow for 16 h at 37°C with 5% CO_2_. The emitted light was measured using a 96-well-plate luminometer (PerkinElmer/Wallac 1420 Victor^3^), and results are expressed in RLU per second. All of these assays were minimally performed in triplicate.

### Bacterial survival assay (3 h).

To measure bacterial survival, different bacteria were exposed to various concentrations of the compounds (as indicated) on their specific growth medium (see the culture conditions described above) for 3 h at 37°C. To perform this assay, all strains were grown overnight in their corresponding agar media. Cell suspensions corresponding to an OD_600_ of 0.1 were subsequently prepared. In parallel, fresh stock solutions of the compounds in DMSO were prepared (100×, with final concentrations being indicated in the different figures), and 1.8 μl of the corresponding solution per well was added to 96-well plates. In each well, 178.2 μl of bacterial suspension were subsequently added. Bacteria were allowed to grow for 3 h at 37°C with 5% CO_2_. After incubation, serial dilutions (to 10^−6^) were performed and 50 μl of each diluted solution was spread on agar plates. After an overnight incubation at 37°C with 5% CO_2_, CFU were enumerated. All of these assays were minimally performed in triplicate.

### MIC determination by agar dilution assay.

To measure bacterial growth inhibition on GCB agar medium, solutions of different bacteria at an OD_600_ of 0.1 were prepared, and 10 μl of serial dilutions was spotted on GCB agar plates containing various concentrations of NaBPh_4_. Each species was grown in its specific growth medium (see the culture conditions described above) for 24 h to 48 h at 37°C. The percent growth values presented in Fig. 3 were calculated by dividing the CFU counts with NaBPh_4_ by the CFU counts with GCB only.

### Determination of bacterial cellular boron and magnesium levels by ICP-MS.

The cellular amount of boron (and magnesium) in *Neisseria* species was determined as reported previously (21), by growing cells overnight on complete GCB medium and subculturing them on several agar plates containing 5 μM (1 μM for N. gonorrhoeae) NaBPh_4_ or NaBF_4_. For this experiment, a no-treatment control (bacteria grown with GCB alone) was also included. After incubation for 8 h, cells were suspended in PBS and centrifuged. Pellets were washed twice with PBS and subjected to a 1-h digestion at 80°C in nitric acid (500 μl of a 65% solution; Sigma-Aldrich), followed by 16 h of incubation at room temperature. The resulting solutions were diluted with water (high-performance liquid chromatography grade; Fisher) to a final concentration of 3% in nitric acid. Samples were analyzed by ICP-MS with a Perkin Elmer NexION 300X at the Department of Chemistry, Université de Montreal (Montréal, Canada). Normalized results were expressed as the calculated ratio of boron (micrograms) to magnesium (micrograms). Experiments were carried out in triplicate.

### Characterization of N. meningitidis mutants with decreased sensitivity.

N. meningitidis isolate LNP24198 was cultured on solid GCB medium for 16 h, and its DNA was extracted with the Qiagen Genomic-tip 100/G kit. The purified DNA was then sequenced with a PacBio Sequel system at Genome Canada (McGill University). The resulting reads were *de novo* assembled with the HGAP4 protocol of the single-molecule real-time (SMRT) Link suite. The consensus sequence was subsequently polished with the resequencing protocol of SMRT Link. The genome was annotated using the webserver DFAST (39).

DNA from four resistant clones was extracted with the QIAamp DNA minikit kit from Qiagen and sequenced with an Illumina MiSeq system at Genome Canada (McGill University). Mutations of the clones relative to the wild-type strain were identified using Snippy version 4.4.0 (https://github.com/tseemann/snippy) with a 60% cutoff value.

For genomic DNA transformation, N. meningitidis isolate LNP24198 was inoculated on a GCB agar plate containing 10 mM MgCl_2_, and 10 μl with ∼500 ng of DNA (from wild-type or mutant strains) was deposited on top of the culture. After a 6-h incubation at 37°C, bacteria were collected and inoculated in selective GCB agar plates containing 17 μM NaBPh_4_. The presence of mutations was assessed using standard Sanger sequencing technology.

### Toxicity of NaBPh_4_ in mice.

To assess the toxicity of NaBPh_4_, one group of five noninfected 6-week-old mice was treated with 100 μl of 1% DMSO as a control (i.p.) and two groups were treated with 100 μl of a solution of NaBPh_4_ in 1% DMSO (i.v. or i.p.). Clinical signs were assessed by scoring the general state of health (activity/lethargy, posture [such as hunched or prostrated], eye abnormality, ruffled fur, and social behaviors) in the cage at the indicated times.

### Mouse infection.

For this experiment, isolate LNP24198 of Neisseria meningitidis, expressing the *luxCDABE* gene under the control of the *porBp* promoter, was used (25, 26). Three groups, each containing five 7-week-old BALB/c mice, were infected with luminescent Neisseria meningitidis. For this, a mixture of 250 μl of bacterial cultures at an OD_600_ of 0.1 (5 × 10^7^ cells/ml) and 100 μl of human transferrin (20 mg/ml) was injected into each mouse. Two hours later, one group was treated with 100 μl of NaBPh_4_ (20 μM) injected by i.v. injection into the tail veins, and one group was treated with the same dose injected by i.p. injection. The remaining five mice were treated with 100 μl of DMSO as controls. NaBPh_4_ was initially prepared at 20 mM in DMSO before being diluted in PBS to reach a final concentration of 20 μM (with 0.1% DMSO). DMSO was diluted in PBS in the same manner to achieve a dilution of 1:1,000. Luminescence was then measured, on the front and back of the mice, at different time points (0, 4, 8, 24, and 48 h postinfection). The light signal was determined for each mouse using the region of interest (ROI) tool of the IVIS Lumina III. This tool measures the total photon count in the ROI. This ROI is a fixed region that represents the entire mouse and has the same surface for all mice. The same infection experiment was reproduced but using an influenza virus coinfection, as described (25, 26). For this, mice were intranasally infected with 25 PFU 7 days prior to N. meningitidis (or isolated mutant) infection.

## Supplementary Material

Supplemental file 1
